# ADGRD1 as a Potential Prognostic and Immunological Biomarker in Non-Small-Cell Lung Cancer

**DOI:** 10.1155/2022/5699892

**Published:** 2022-11-22

**Authors:** Meiwen Lv, Xuelian Li, Wen Tian, He Yang, Baosen Zhou

**Affiliations:** ^1^Department of Clinical Epidemiology, The First Hospital of China Medical University, 155 Nanjing Street, Heping District, Shenyang 110001, China; ^2^Department of Epidemiology, School of Public Health of China Medical University, Shenyang 110122, China

## Abstract

ADGRD1 (GPR133), an adhesion G protein-coupled receptor (GPCR), has been linked to cancer. However, the prognostic value and regulatory function within non-small-cell lung cancer (NSCLC) is still unclear. This work adopted various bioinformatics methods, including publicly available databases as well as real-time PCR (RT-PCR), for detecting ADGRD1 expression level and investigating the correlation between ADGRD1 expression level and prognosis, tumor mutational burden (TMB), microsatellite instability (MSI), immune infiltrating cells, immune-related genes, and targeted regulation mechanisms in NSCLC. According to the results, ADGRD1 expression decreased within NSCLC, which might be the factor predicting prognosis of NSCLC. Meanwhile, ADGRD1 showed significant correlation with TMB and MSI, respectively, as well as immune cell infiltrating levels in lung adenocarcinoma (LUAD), which were primarily linked to macrophage M1, mast cell resting, T cell CD4 memory activated, and T cell CD4 memory resting and were associated with mast cell activated and mast cell resting in lung squamous cell carcinoma (LUSC). The most promising upstream regulation pathways of ADGRD1 were likely miR-142-5p, miR-93-5p, and miR-17-5p, which were overexpressed and associated with poor prognosis in NSCLC. ADGRD1 and immune-related genes correlated with ADGRD1 were shown to be enriched in “positive regulation of leukocyte activation,” “external side of plasma membrane,” “receptor ligand activity,” and “cytokine-cytokine receptor interaction” pathways. ADGRD1 expression and regulation may be critical in determining NSCLC prognosis.

## 1. Introduction

Cancer represents a major factor causing global mortality, endangering human health and life safety. Lung and bronchus cancer accounts for 235,760 newly diagnosed and 131880 death cases in the United States by 2021, making it the main cause of death in patients and the survival rate at 5 years is just about 21% [1]. NSCLC accounts for roughly 85% of lung cancers, with LUAD and LUSC being the most prevalent subtypes [2]. In recent years, many reports on lung cancer markers related to prognosis and immunity suggest that these markers are critical in understanding the occurrence and development of NSCLC and immunotherapy.

The GPCR adhesion family is extensively expressed in human tissues and important in human physiological processes. GPCRs are linked to several diseases [3–5], implying that GPCRs have a critical effect on onset, development, and therapy, including cancer. ADGRD1, an adhesion G protein-coupled receptor, is a key modulator of signal transduction [6] and has been linked to cancer [7]. However, the mechanism of ADGRD1 expression in NSCLC carcinogenesis and the prognostic and immunological roles of ADGRD1 remain unknown.

In a nutshell, TMB indicates the overall mutation number within tumors [8, 9]. TMB and microsatellite instability-high (MSI-H) levels can be viewed as predictive biomarkers participating in tumor immunotherapy [10, 11]. The tumor microenvironment typically contains immune and stromal cells, and immune score is a powerful predictor of clinical outcomes. It has been observed that numerous immune infiltrating cells exist within the tumor microenvironment and has an important effect on tumor growth [12], which is important for tumor immunotherapy. Furthermore, the role of signaling pathways in tumor formation in the tumor microenvironment should not be overlooked. According to research, the Notch signaling pathway participates in numerous life activities of cancer cells in the tumor microenvironment, and it has a significant effect on cancer occurrence and development [13].

Previously, biological function of ADGRD1 in different human cancers has not been completely explored and only a few researches on ADGRD1 analysis in NSCLC have been published. Multiple databases were used in this study for exploring the prognostic significance of ADGRD1 within pan-cancer. In addition, ADGRD1 expression and the link between TMB, MSI, and immune infiltrating cells were investigated. Furthermore, the relationship between ADGRD1 and immune genes was investigated, signaling pathways with relevant immune genes. Finally, the microRNAs controlled by ADGRD1 were predicted, and the relationship between these miRNAs and NSCLC prognosis was examined.

## 2. Materials and Methods

### 2.1. Analysis of ADGRD1 Expression and Data Sources

RNA sequences, somatic mutations, and survival data associated with a total of 33 cancers were downloaded from UCSC Xena (http://xena.ucsc.edu/), a convenient database for obtaining TCGA database data. The inclusion criteria were as follows: completed ADGRD1 mRNA sequencing data, completed somatic mutations data, and detailed clinical follow-up information. R packages “plyr” and “ggpubr” were applied to draw the boxplot of gene expression sequences. Pan-cancer ADGRD1 expression analysis was also performed by GEPIA2 (http://gepia2.cancer-pku.cn/) database which contained TCGA and GTEx data.

### 2.2. Prognostic Significance of Gene ADGRD1 within Pan-Cancer

By adopting the Cox regression and the Kaplan-Meier curves, ADGRD1 expression and prognostic associated data were matched to examine the association between ADGRD1 and overall survival (OS), disease-specific survival (DSS), disease-free interval (DFI), and progression-free interval (PFI). The Cox regression analysis evaluated the association between survival time and survival status by considering gene expression as a continuous variable. Concurrently, hazard ratio (HR) with the 95% confidence interval was considered. An HR value greater than one indicated that ADGRD1 was a risk factor rather than a protective factor in this cancer. The Kaplan-Meier approach was utilized to classify cases as the high-expression or low-expression group according to their median level. They analyzed the different OS of both the expression groups. The R packages “survival” and “survminer” were used to draw the Kaplan-Meier curves.

### 2.3. Analysis of TMB and MSI of ADGRD1 Expression in Pan-Cancer

TMB is the overall mutation number within DNA carried via cancer cells. MSI indicates contaminant nucleotide gain/loss in repetitive DNA fragments. MSI score could be analyzed based on the data of TCGA. The radar map containing the correlation between ADGRD1 expression level and TMB or MSI was performed by “fmsb” R package.

### 2.4. Correlation of ADGRD1 Expression with Tumor Immune Microenvironment and Tumor-Infiltrating Immune Cells within Pan-Cancer

This work adopted ESTIMATE algorithm to determine immune, stromal, and ESTIMATE scores with “limma” and “estimate” packages in R software, for predicting the purity of different cell types within the tumor microenvironment. The estimation of immune infiltrating cells in NSCLC was analyzed using CIBERSORT [14]. Coexpression analysis of ADGRD1 level with tumor immune microenvironment and tumor-infiltrating immune cells was showed by “ggplot2,” “ggpubr,” and “ggExtra” packages.

### 2.5. Correlation Analysis between ADGRD1 Expression and Immune-Related Genes

We downloaded 1793 immune-related genes from ImmPort database (https://www.immport.org/home) for further screening genes significantly coexpressed with ADGRD1 in both LUAD and LUSC (*P* < 0.001 and |*R*| > 0.2 as the cut-off criterion). After screening, Gene Ontology (GO) and Kyoto Encyclopedia of Genes and Genomes (KEGG) analyses were performed by ADGRD1 and screened genes. R packages “org.Hs.eg.db,” “clusterProfiler,” “enrichplot,” and “ggplot2” were utilized to show the result of GO and KEGG analyses with the cut-off threshold *P* value = 0.05 and *q* value = 1.

### 2.6. Prediction of ADGRD1-Interacted MicroRNAs Related to Prognosis

To predict upstream microRNAs that could regulate ADGRD1 expression, starBase database (https://starbase.sysu.edu.cn/) was used. MicroRNA expression and clinical information from NSCLC cases were downloaded in TCGA (https://portal.gdc.cancer.gov/). The inclusion criteria were as follows: (1) completed microRNA sequencing data and detailed clinical follow-up information; (2) miRNAs from starBase database with the screening threshold as programNum > = 2 and targeted ADGRD1. The exclusion criteria were as follows: miRNAs not negatively correlated with ADGRD1. The “survival” package of R language was used in identifying the prognostic microRNAs. And the cut-off criteria of differential expression were |log_2_fold change| = 1 and diffPval = 0.05.

### 2.7. Cell Culture and RT-PCR

This work cultivated the BEAS-2B normal human lung epithelial cells within RPMI-DMEM that contained 20% FBS (fetal calf serum), whereas NCI-H1299 human NSCLC cells within RPMI-1640 medium that contained 10% FBS. Following Takara RR820A kit instruction, RT-PCR was carried out, with U6 being the endogenous control. Takara Biomedical Technology (Shenyang, China) designed and synthesized ADGRD1 primer. Method of 2^−△△Ct^ was calculated to the relative transcription level of ADGRD1. The sequences of primers were as follows:

### 2.8. Data Extraction Based on the Human Protein Atlas (HPA)

The HPA project (https://www.proteinatlas.org/) covers protein levels in tissues, cancers, and normal cells. IHC was applied to validate ADGRD1 expression levels within normal lung and lung tumor tissues.

### 2.9. Statistical Analysis

The Wilcoxon test was applied to calculate the difference between normal tissues and tumor tissues about ADGRD1 expression with the cut-off standard of *P* = 0.05. The Cox regression and Kaplan-Meier methods using log-rank tests were utilized to evaluate whether ADGRD1 predicted the prognosis of pan-cancer (*P* = 0.05). All correlation analyses were carried out by the Spearman method (*P* < 0.001 and |*R*| > 0.2 as cut-off values).

## 3. Results

### 3.1. ADGRD1 Expression Level in Pan-Cancer

**Figure 1:**
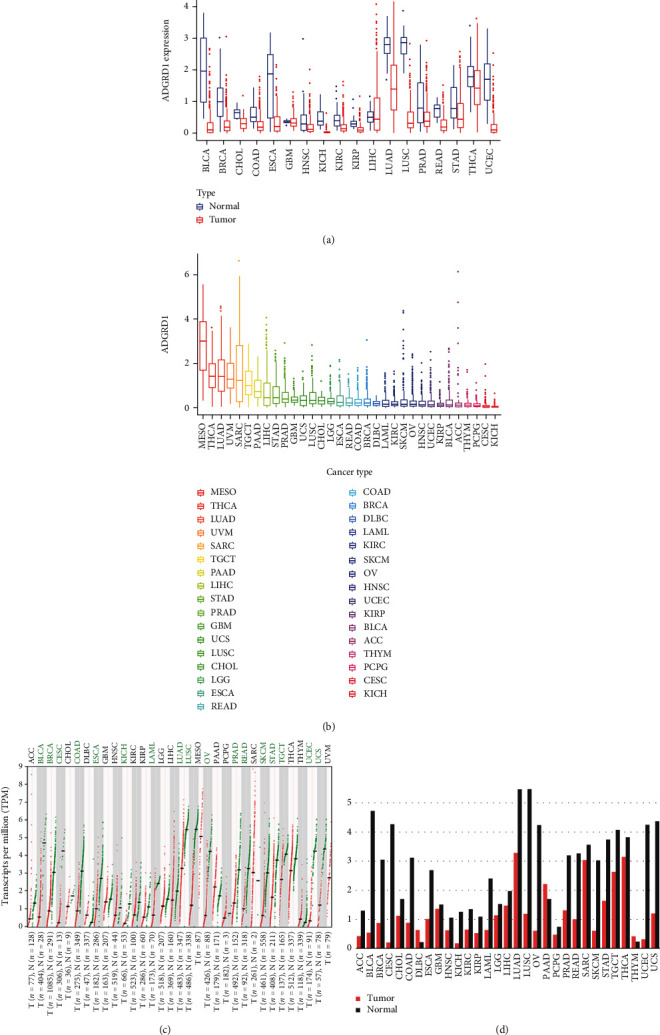
ADGRD1 expression levels in pan-cancer. (a) The expression levels of ADGRD1 in diverse cancers from TCGA data (^∗^*P* < 0.05, ^∗∗^*P* < 0.01, ^∗∗∗^*P* < 0.001). (b) The expression levels of ADGRD1 in diverse tumor tissues from TCGA data. (c) The dot plot of ADGRD1expression in different cancers from TCGA and GTEx data. Each red dots represent expression of tumor tissue and each green dots represent expression of normal tissue. The green font indicates that ADGRD1 is significantly downregulated in tumor tissue compared to normal tissue. (d) The bar plot of ADGRD1 expression in different cancers from TCGA and GTEx data. The height of bar represents the median expression of each sample.

First, we analyzed the expression of gene ADGRD1 in different cancers and found that gene ADGRD1 was differentially low-expressed in bladder urothelial carcinoma (BLCA), breast invasive carcinoma (BRCA), cholangiocarcinoma (CHOL), colon adenocarcinoma (COAD), esophageal carcinoma (ESCA), head and neck squamous cell carcinoma (HNSC), kidney chromophobe (KICH), kidney renal clear cell carcinoma (KIRC), kidney renal papillary cell carcinoma (KIRP), LUAD, LUSC, prostate adenocarcinoma (PRAD), rectum adenocarcinoma (READ), stomach adenocarcinoma (STAD), thyroid carcinoma (THCA), and uterine corpus endometrial carcinoma (UCEC) between normal tissue and tumor tissue data from TCGA (Figure 1(a)). Meanwhile, the data was also used to evaluate ADGRD1 expression in 33 tumor tissues, and the expression level of gene ADGRD1 in LUAD and LUSC was ranked third and thirteenth, respectively (Figure 1(b)). Furthermore, we applied GEPIA-combined data from TCGA and GTEx database for analysis to verify this result (Figure 1(c)), which indicated that ADGRD1 was expressed abnormally in BLCA, BRCA, cervical squamous cell carcinoma and endocervical adenocarcinoma (CESC), COAD, ESCA, KICH, acute myeloid leukemia (LAML), LUAD, LUSC, ovarian serous cystadenocarcinoma (OV), PRAD, READ, skin cutaneous melanoma (SKCM), STAD, testicular germ cell tumors (TGCT), UCEC, and uterine carcinosarcoma (UCS). As for the results showed in Figure 1(d), ADGRD1 expression of NSCLC was dramatically downregulated in tumor samples compared with paired normal tissues, which had the highest ADGRD1 mRNA levels across multiple normal tissues.

### 3.2. Prognostic Significance of Gene ADGRD1 in Pan-Cancer

**Figure 2:**
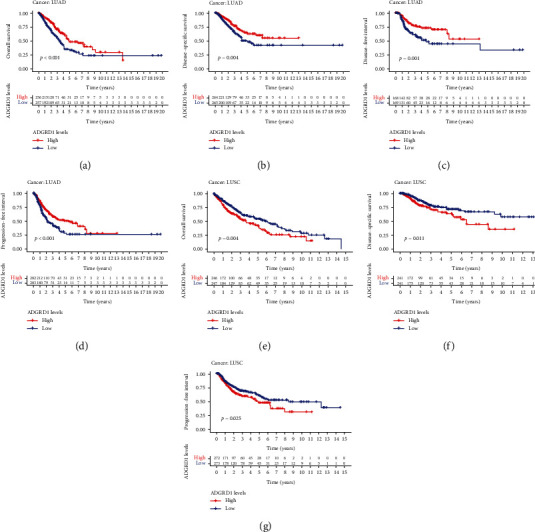
Kaplan-Meier survival curves of OS, DSS, DFI, and PFI in NSCLC. (a) OS analysis of ADGRD1 in LUAD. (b) DSS analysis of ADGRD1 in LUAD. (c) DFI analysis of ADGRD1 in LUAD. (d) PFI analysis of ADGRD1 in LUAD. (e) OS analysis of ADGRD1 in LUSC. (f) DSS analysis of ADGRD1 in LUSC. (g) PFI analysis of ADGRD1 in LUSC.

First, we used the Cox regression to analyze the impact of ADGRD1 expression among OS, DSS, DFI, and PFI in pan-cancer (Table 1). The results showed that ADGRD1 played an important role in the prognosis of twelve cancers, which included adrenocortical carcinoma (ACC), COAD, KIRP, LAML, brain lower grade glioma (LGG), liver hepatocellular carcinoma (LIHC), LUAD, LUSC, mesothelioma (MESO), READ, sarcoma (SARC), and STAD. The expression of ADGRD1 was a protective factor in LUAD (OS: HR = 0.776, 95% CI from 0.663 to 0.908, *P* = 0.002; DSS: HR = 0.777, 95CI% from 0.650 to 0.928, *P* = 0.005; DFI: HR = 0.743, 95% CI from 0.599 to 0.922, *P* = 0.007; PFI: HR = 0.846, 95% CI from 0.741 to 0.966, *P* = 0.014), while a risk factor in LUSC (OS: HR = 1.483, 95% CI from 1.092 to 2.012, *P* = 0.011; DSS: HR = 1.271, 95CI % from 1.007 to 1.605, *P* = 0.043). Kaplan-Meier survival curves also showed that ADGRD1 expression was correlated with the prognosis in LUAD (OS: *P* < 0.001; DSS: *P* = 0.004; DFI = 0.001; PFI: *P* < 0.001) and LUSC (OS: *P* = 0.004; DSS: *P* = 0.011; PFI: *P* = 0.025); meanwhile, the significant results were displayed in Figure 2.

### 3.3. Correlation of ADGRD1 Expression with TMB and MSI in Pan-Cancer

**Figure 3:**
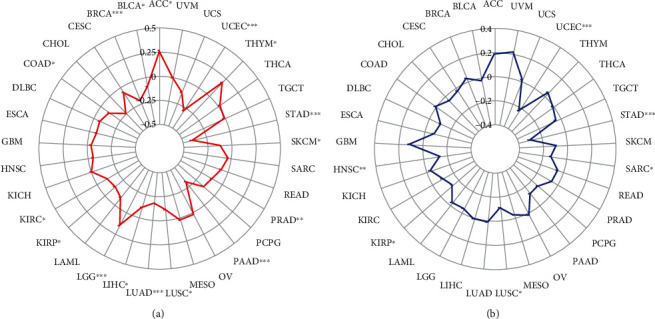
Correlation between ADGRD1 expression and TMB, MSI across cancers. (a) The radar chart showed the correlation between ADGRD1 expression and TMB in diverse cancers (^∗^*P* < 0.05, ^∗∗^*P* < 0.01, ^∗∗∗^*P* < 0.001). (b) The radar chart showed the correlation between ADGRD1 expression and MSI in multiple cancers (^∗^*P* < 0.05, ^∗∗^*P* < 0.01, ^∗∗∗^*P* < 0.001).

TMB and MSI have been regarded as meaningful predictors of immune response. In the analysis of the association between ADGRD1 expression and TMB, MSI in pan-cancer is essential. As shown in Figure 3(a), ADGRD1 expression was positively associated with TMB in ACC (*P* = 0.019), LGG (*P* = 0.001) and thymoma (THYM, *P* = 0.038), while negatively related to TMB in BLAC (*P* = 0.048), BRCA (*P* < 0.001), COAD (*P* = 0.019), KIRC (*P* = 0.025), KIRP (*P* = 0.016), LIHC (*P* = 0.029), LUAD (*P* < 0.001), LUSC (*P* = 0.012), pancreatic adenocarcinoma (PAAD, *P* < 0.001), PRAD (*P* = 0.007), SKCM (*P* = 0.010), STAD (*P* < 0.001), and UCEC (*P* < 0.001). Furthermore, we calculated the correlation between ADGRD1 expression and MSI across cancers (Figure 3(b)). ADGRD1 expression was negatively related to MSI in HNSC (*P* = 0.001), KIRP (*P* = 0.022), LUSC (*P* = 0.020), SARC (*P* = 0.042), STAD (*P* < 0.001), and UCEC (*P* < 0.001). According to the results, the expression of ADGRD1 was both correlated with TMB and MSI in LUSC, however, only significantly correlated with TMB in LUAD.

### 3.4. Immunological Role of Gene ADGRD1

**Figure 4:**
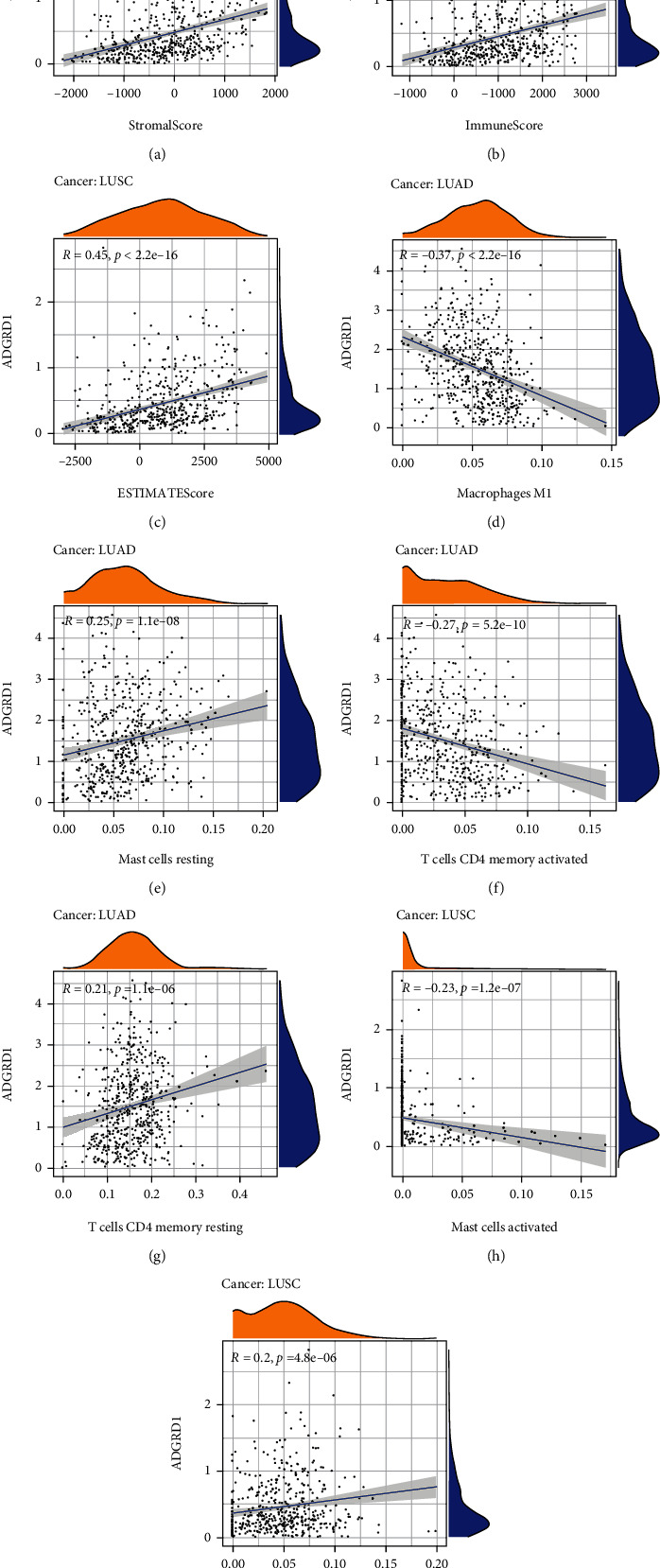
Correlation of ADGRD1 expression with tumor immune microenvironment and immune cell infiltration in NSCLC. (a) Correlation analysis between ADGRD1 expression in LUSC and StromalScore. (b) Correlation analysis between ADGRD1 expression in LUSC and ImmuneScore. (c) Correlation analysis between ADGRD1 expression in LUSC and ESTIMATEScore. (d–g) Correlation between ADGRD1 expression and immune infiltrating cells in LUAD. (h–i) Correlation between ADGRD1 expression and immune infiltrating cells in LUSC.

We explore the association between tumor immune microenvironment, immune infiltrating cells, and ADGRD1 expression. The ESTIMATE algorithm including the StromalScore, ImmuneScore, and ESTIMATEScore was utilized to evaluate the association between ADGRD1 expression and different types of infiltrating cells in the tumor immune microenvironment across cancers and the results were shown in Table S1. As for LUSC, ADGRD1 expression was positively correlated with all scores (Figures 4(a)–4(c)). However, the expression of ADGRD1 was not significantly correlated with any score in LUAD (StromalScore, *P* = 0.567; ImmuneScore, *P* = 0.007; ESTIMATEScore, *P* = 0.190). Varieties of immune infiltrating cells were contained in the tumor microenvironment. Thus, we calculated the association between ADGRD1 expression and immune cell infiltration levels in NSCLC. In LUAD, ADGRD1 was mainly related to 4 immune cells (macrophage M1, mast cell resting, T cell CD4 memory activated, and T cell CD4 memory resting) in LUAD and associated with 2 immune cells (mast cell activated and mast cell resting) in LUSC (Figures 4(d)–4(i)). The significant correlation of 22 immune cells with other cancers was performed in Figure S1.

### 3.5. Coexpression of Immune-Related Genes with ADGRD1 and Associated Pathway Analysis in NSCLC

**Figure 5:**
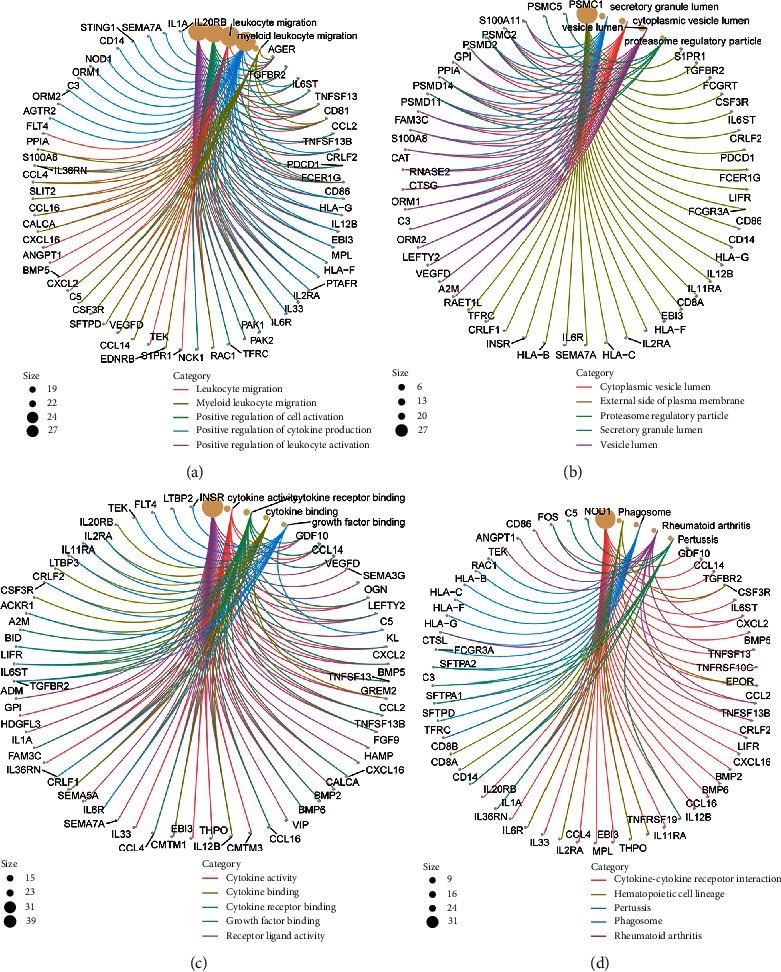
GO and KEGG analyses of ADGRD1 and immune-related genes. (a) Circle graph of BP category in GO functional annotation analysis. The size means the number of genes. (b) Circle graph of CC category in GO functional annotation analysis. The size means the number of genes. (c) Circle graph of MF category in GO functional annotation analysis. The size means the number of genes. (d) Circle graph of KEGG pathway analysis. The size means the number of genes.

We used ImmPort database to obtain the immunologically relevant list with a total of 1793 genes. After analysis, only 149 immune-related genes had significant differences correlated with ADGRD1 in NSCLC (Table 2). Through the GO analysis of the eligible 149 genes and ADGRD1, the results were shown in Figures 5(a)–5(c), implying that the total 150 genes were enriched in biological process (BP) category with “positive regulation of leukocyte activation”, “positive regulation of cell activation”, and “leukocyte migration”, meanwhile, “external side of plasma membrane”, “secretory granule lumen”, and “cytoplasmic vesicle lumen” in cellular component (CC) category, additionally including “receptor ligand activity”, “cytokine activity”, and “cytokine receptor binding” in molecular function (MF) category. Regarding KEGG, “cytokine−cytokine receptor interaction”, “hematopoietic cell lineage”, and “phagosome” were enriched pathways (Figure 5(d)).

### 3.6. Predictive Analysis of Intersected MicroRNAs of ADGRD1

**Figure 6:**
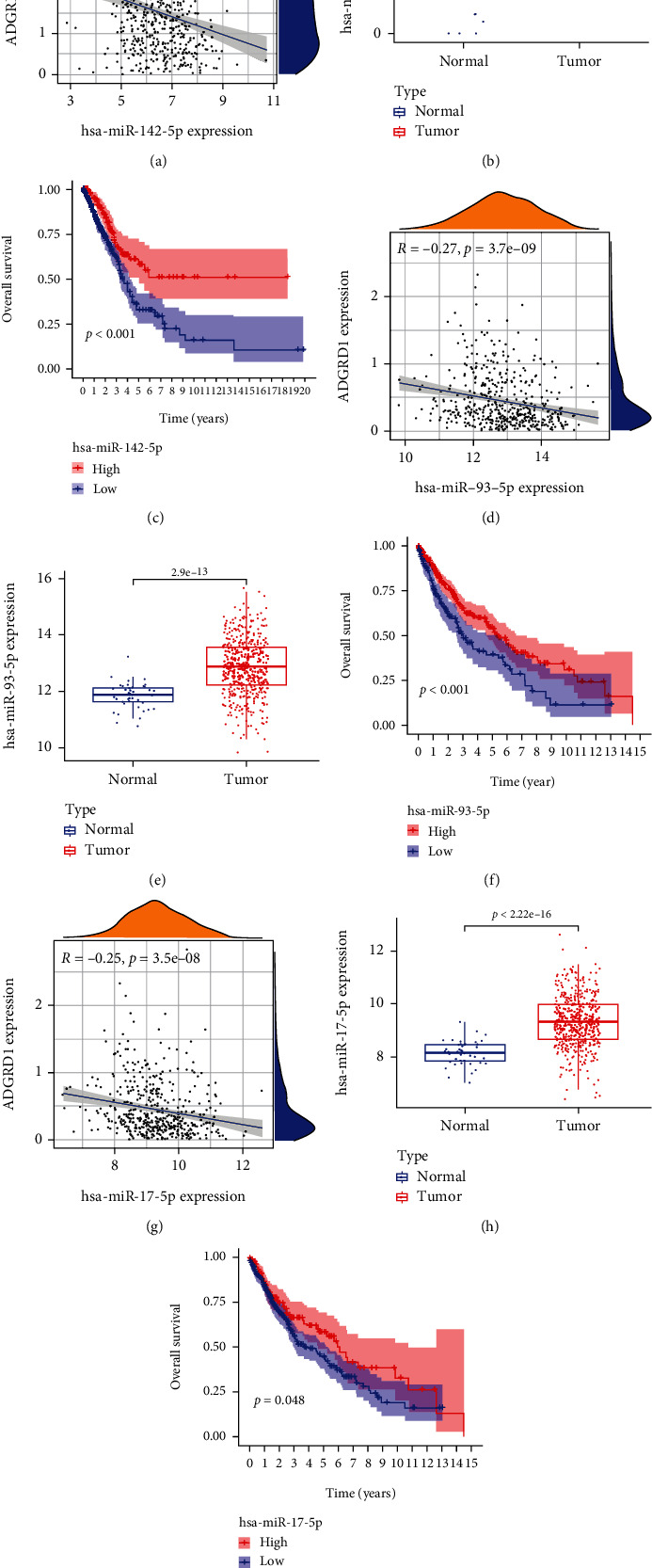
Correlation, expression, and survival analysis for intersected microRNAs of ADGRD1 in NSCLC. (a) Correlation of miR-142-5p and ADGRD1 expression in LUAD. (b) Expression of miR-142-5p in LUAD. (c) Survival analysis of miR-142-5p in LUAD. (d) Correlation of miR-93-5p and ADGRD1 expression in LUSC. (e) Expression of miR-93-5p in LUSC. (f) Survival analysis of miR-93-5p in LUSC. (g) Correlation of miR-17-5p and ADGRD1 expression in LUSC. (h) Expression of miR-17-5p in LUSC. (i) Survival analysis of miR-17-5p in LUSC.

We used starBase database to predict the miRNAs targeting ADGRD1, and 20 upstream miRNAs were discovered. The inclusion criteria of miRNAs were significantly correlated with ADGRD1, differentially expressed, and the prognosis was statistically significant in LUAD or LUSC. After a series of screening criteria, as shown in Table 3, only miR-142-5p was significantly negatively correlated with ADGRD1 in LUAD, which were both differentially expressed and associated with prognosis (Figure 6(a)–6(c)). Meanwhile, miR-93-5p and miR-17-5p were the same correlated with ADGRD1 in LUSC (Figure 6(d)–6(i)).

### 3.7. Validation of ADGRD1 Expression in Cell Lines

**Figure 7:**
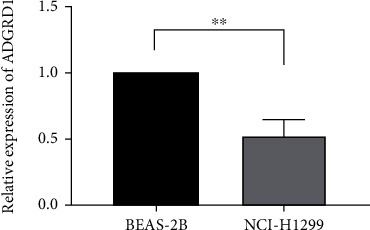
The expression levels of ADGRD1 in BEAS-2B and NCI-H1299 cells (^∗^*P* < 0.05, ^∗∗^*P* < 0.01, ^∗∗∗^*P* < 0.001).

Compared with BEAS-2B cells, the expression level of ADGRD1 mRNA was lower in NCI-H1299 cells, which is consistent with the prior research (Figure 7).

### 3.8. Validation of the Protein Expression Level by IHC of Gene ADGRD1

**Figure 8:**
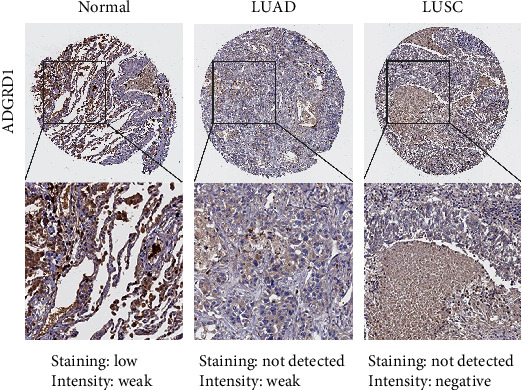
The immunohistochemical results of ADGRD1 in normal, LUAD, and LUSC tissues from the HPA.

As shown in Figure 8, the results from the HPA revealed the lower expression of ADGRD1 protein in LUAD and LUSC tissues compared to normal tissues.

## 4. Discussion

Pan-cancer analysis is of great significance for comparing the role of the same gene in different cancers and provides a new direction for new tumor biomarkers, understanding the mechanism of tumorigenesis and development from the prevention and treatment of tumors. The same gene has the same or different effects on different tumorigenesis, mutations, tumor microenvironment, and copy number changes, which can be vital and helpful to diagnose and treat different tumors. CD161 pan-cancer analysis suggests that CD161 is the possible antitumor biomarker to develop the novel agents [15]. In addition, PDIA3 is the pan-cancer gene and a factor independently predicting prognosis in the prognostic outcome of KIRP and KICH cases; meanwhile, it significantly affects immunotherapeutic response among THYM, LGG, and READ cases [16]. As revealed by pan-cancer analysis, POU5F1 can be used to diagnose and predict the prognosis of different cancers in particular functional carcinogenicity of LIHC [17]. In this research, we investigated the underlying molecular mechanism of ADGRD1 across cancers; downregulation of ADGRD1 expression in NSCLC was significantly correlated with prognosis, TMB, MSI, tumor microenvironment, and immune infiltrating cells. This provides new ideas and directions for strengthening the understanding of ADGRD1 in various cancers and revealing it as a new clinical biomarker for prognosis and immunotherapy. In addition, the analysis of ADGRD1 in NSCLC may bring hope for strengthening the prevention of NSCLC, improving the treatment of NSCLC patients, and enhancing the survival rate of patients.

According to this research, ADGRD1 expression in pan-cancer was investigated using UCSC Xena database and GEPIA, and it was discovered that ADGRD1 expression level was relatively high in NSCLC cases compared to other cancers, particularly in NSCLC tissues, where ADGRD1 expression remarkably decreased compared with normal tissues. RT-PCR revealed that, compared to normal lung epithelial cells, ADGRD1 expression declined within NSCLC cells; meanwhile, immunohistochemical results of ADGRD1 obtained from the HPA revealed that compared to normal tissues, ADGRD1 protein expression decreased within NSCLC tissues, conforming to prior findings. According to our pan-cancer analysis, ADGRD1 had a critical effect on prognosis of different cancers. Few studies have reported the role of ADGRD1 in NSCLC prognosis. In this study, ADGRD1 downexpression was linked to a worse prognosis in LUAD and a better prognosis in LUSC, implying that ADGRD1 could be a viable prognostic biomarker in NSCLC.

TMB has been demonstrated to be an independent predictor of immunotherapy response in various cancers, as well as a predictor of patient clinical outcomes [18, 19]. MSI is an important clinical tumor marker induced by functional abnormalities in DNA mismatch repair [20]. MSI has been linked to various cancers, including endometrial, colorectal, and prostate cancer [21–23]. According to this study, ADGRD1 expression is related to TMB in 16 cancer types, including LUAD and LUSC, and MSI in 6 cancer types, including LUSC. The findings suggested that abnormal ADGRD1 expression could be a useful biomarker for tumor detection and treatment and linked to MSI and TMB.

The tumor microenvironment has become a popular issue in tumor research. Immune cells have critical effects on tumor microenvironment [24]. Plasma complement factor B expression was linearly associated with macrophage M1 cells in the tumor microenvironment of thyroid carcinoma [25]. In hepatocellular carcinoma, resting mast cells in the tumor microenvironment were adversely linked with PD-L1 mRNA expression [26]. There should be a positive relationship between CD52 expression and activated memory CD4+ T cells in breast cancer [27]. CCR4, CCR8, and P2RY14 expressions were positively linked with T cells CD4+ memory resting in head and neck squamous cell cancer [28]. Activated mast cells and resting mast cells were associated with C3 in colorectal adenocarcinoma [29]. As a result of the preceding, it might be concluded that various immune cell contents in the tumor microenvironment of different cancers were related to the expression of different genes. However, there have been few reports linking ADGRD1 to the tumor microenvironment. ADGRD1 expression was connected with macrophage M1, mast cell resting, T cell CD4 memory activated, and T cell CD4 memory resting in LUAD and be associated with mast cell activated and mast cell resting in LUSC.

Abnormal immune-related gene expression is strongly associated with immune infiltrating cells in various ways, making it a potential diagnostic and therapeutic target that provides new avenues for studying tumor molecular mechanisms of tumors [30–32]. Additionally, tumor-related genes influence various biological processes involved in tumor formation and treatment though activating distinct signaling pathways [33–35]. In this study, ADGRD1 was significantly linearly correlated with 149 immune-related genes in NSCLC, and for GO functional annotation analysis, these genes were most enriched in terms “positive regulation of leukocyte activation”, “positive regulation of cell activation” , and “leukocyte migration” in BP category, “external side of plasma membrane”, “secretory granule lumen”, and “cytoplasmic vesicle lumen” in CC category, and “receptor ligand activity”, “cytokine activity”, and “cytokine receptor binding” in MF category. According to KEGG pathway analysis, the most enriched pathways of ADGRD1 and associated immune-related genes in NSCLC were “cytokine−cytokine receptor interaction,” “hematopoietic cell lineage,” and “phagosome.” The findings imply that these genes possibly have critical effects on NSCLC cancer pathogenesis, progression, and tumor therapy through revealing tumor molecular life processes via these pathways.

By understanding tumor-related biological pathways, microRNAs have provided critical hints for developing active diagnostic and therapeutic targets or unique prognostic biomarkers. The miR-6845-5p/miR-4455-ADGRD1 pathway was related to gastric cancer incidence [36]. miR-142-5p overexpression may increase breast cancer proliferation, invasion, and migration when SORBS1 is targeted [37]. Increased apoptosis and decreased recurrence rate are associated with increased radio-sensitivity of breast cancer cells when miR-93-5p is overexpressed, implying the role of miR-93-5p may act as a potential therapeutic target for breast cancer [38]. miR-17-5p may have an important effect on pathogenesis of triple-negative breast cancer, and its increased expression is associated with prognosis, implying the role of miR-17-5p as the therapeutic target for triple-negative breast cancer [39]. According to our results, miR-142-5p was connected with prognosis LUAD-targeted ADGRD1, but miR-93-5p and miR-17-5p-targeted ADGRD1 were found to be associated with LUSC prognosis.

However, this study has several drawbacks. First, bioinformatics analysis of ADGRD1 was performed using several databases, which may introduce systematic bias. Second, although RT-PCR technique was involved for verifying ADGRD1 expression within NSCLC, it would be more precise to carry out in vitro/in vivo experiments and even clinical research to prove the prognostic role of ADGRD1in this study. Third, despite the expression of ADGRD1 was detected to be associated with tumor-infiltrating immune cells and prognosis of patients, we were unable to confirm the exact mechanism of the prognostic features. Therefore, future prospective studies focusing on ADGRD1 expression and immune infiltrating cells may be needed to solve this issue.

## 5. Conclusion

In summary, we found that ADGRD1 showed relationship to the clinical outcomes, TMB, MSI, and immune infiltrating cells in multiple cancers. In NSCLC, ADGRD1 expression was lowered and significantly associated with prognosis, TMB, MSI, tumor microenvironment, and immune infiltrating cells. Additionally, the upstream miRNAs of ADGRD1, ADGRD1 associated immune-related genes, together with ADGRD1 and its associated immune-related genes enriched pathways were defined in NSCLC. As a result, the expression and the mechanism regulation of ADGRD1 might be the prognostic markers in NSCLC.

## Figures and Tables

**Algorithm 1:**
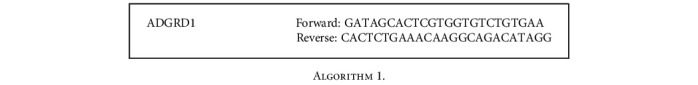


**Table 1:** Cox regression of ADGRD1 expression among OS, DSS, DFI, and PFI in 33 human cancers.

Cancer	OS				DSS				DFI				PFI			
	HR	HR.95 L	HR.95H	*P* value	HR	HR.95 L	HR.95H	*P* value	HR	HR.95 L	HR.95H	*P* value	HR	HR.95 L	HR.95H	*P* value
ACC	1.394	1.093	1.778	0.008	1.413	1.107	1.804	0.006	1.725	0.509	5.845	0.381	1.384	1.104	1.736	0.005
BLCA	1.187	0.898	1.568	0.229	1.207	0.930	1.566	0.157	0.511	0.162	1.612	0.252	1.010	0.793	1.287	0.933
BRCA	0.975	0.565	1.683	0.929	1.428	0.948	2.151	0.089	0.967	0.564	1.661	0.905	1.026	0.712	1.478	0.890
CESC	0.784	0.171	3.600	0.754	1.396	0.546	3.572	0.486	2.080	0.761	5.682	0.153	1.395	0.640	3.039	0.402
CHOL	1.699	0.244	11.844	0.593	1.334	0.203	8.784	0.764	1.677	0.183	15.329	0.647	0.417	0.066	2.617	0.351
COAD	2.250	1.109	4.562	0.025	2.077	0.969	4.450	0.060	1.049	0.238	4.618	0.949	1.536	0.855	2.758	0.151
DLBC	0.285	0.001	134.504	0.689	0.583	1.239*E* − 04	2747.164	0.901	0.478	1.502*E* − 05	15211.505	0.889	0.157	0.001	36.696	0.506
ESCA	0.615	0.320	1.183	0.145	0.915	0.502	1.667	0.771	1.053	0.580	1.912	0.866	0.772	0.497	1.200	0.251
GBM	1.625	0.806	3.277	0.175	2.248	0.885	5.706	0.088	—	—	—	—	0.906	0.418	1.963	0.802
HNSC	0.966	0.572	1.631	0.898	0.921	0.529	1.604	0.771	1.375	0.487	3.886	0.548	0.881	0.544	1.426	0.605
KICH	5.022	0.050	508.254	0.493	2.099	0.269	16.387	0.480	3.420	0.120	97.860	0.472	2.041	0.400	10.412	0.391
KIRC	1.074	0.523	2.208	0.845	0.975	0.463	2.053	0.948	8.558	1.518	48.261	0.015	1.566	0.901	2.722	0.112
KIRP	7.254	2.246	23.435	0.001	9.247	2.849	30.014	2.134*E* − 04	8.121	1.094	60.305	0.041	5.595	1.617	19.352	0.007
LAML	1.987	1.117	3.536	0.019	—	—	—	—	—	—	—	—	—	—	—	—
LGG	2.858	1.675	4.877	1.177*E* − 04	3.489	2.023	6.016	6.982*E* − 06	0.230	0.015	3.505	0.290	2.594	1.579	4.259	1.656*E* − 04
LIHC	1.283	1.066	1.544	0.008	1.423	1.147	1.765	0.001	1.034	0.844	1.267	0.746	1.094	0.921	1.299	0.306
LUAD	0.776	0.663	0.908	0.002	0.777	0.650	0.928	0.005	0.743	0.599	0.922	0.007	0.846	0.741	0.966	0.014
LUSC	1.483	1.092	2.012	0.011	1.271	1.007	1.605	0.043	1.208	0.895	1.631	0.217	1.123	0.927	1.359	0.236
MESO	0.757	0.645	0.889	0.001	0.774	0.626	0.958	0.018	0.723	0.415	1.257	0.250	0.851	0.707	1.023	0.085
OV	1.174	0.841	1.640	0.346	1.196	0.840	1.703	0.321	1.360	0.859	2.153	0.189	1.283	0.931	1.769	0.128
PAAD	0.691	0.459	1.040	0.077	0.783	0.503	1.221	0.281	0.899	0.409	1.979	0.792	0.763	0.517	1.126	0.173
PCPG	0.025	3.276*E* − 06	184.170	0.416	0.214	8.058*E* − 05	566.661	0.701	0.005	3.554*E* − 09	5882.214	0.453	6.505	0.834	50.755	0.074
PRAD	0.191	0.017	2.146	0.180	0.012	3.800*E* − 05	3.807	0.132	0.347	0.116	1.035	0.058	0.548	0.317	0.948	0.032
READ	3.371	1.297	8.760	0.013	1.606	0.425	6.069	0.485	2.071	0.266	16.092	0.487	1.375	0.570	3.319	0.479
SARC	0.827	0.714	0.958	0.011	0.812	0.689	0.959	0.014	0.776	0.652	0.925	0.005	0.816	0.723	0.921	0.001
SKCM	0.874	0.617	1.239	0.450	0.959	0.673	1.366	0.815	—	—	—	—	0.887	0.664	1.184	0.414
STAD	1.374	1.069	1.766	0.013	1.530	1.122	2.085	0.007	1.713	1.108	2.650	0.016	1.385	1.063	1.806	0.016
TGCT	1.179	0.258	5.386	0.832	1.063	0.200	5.665	0.943	0.986	0.582	1.672	0.960	0.968	0.602	1.555	0.892
THCA	1.641	0.816	3.299	0.165	2.260	0.909	5.623	0.079	0.872	0.526	1.445	0.594	0.949	0.664	1.356	0.774
THYM	1.181	0.088	15.875	0.900	0.401	0.008	20.633	0.650	—	—	—	—	3.141	1.484	6.648	0.003
UCEC	0.840	0.418	1.690	0.626	0.673	0.319	1.418	0.297	0.855	0.444	1.647	0.639	0.910	0.595	1.391	0.662
UCS	0.743	0.283	1.950	0.547	0.781	0.298	2.047	0.615	0.674	0.099	4.573	0.687	0.853	0.359	2.027	0.718
UVM	1.716	0.957	3.076	0.070	1.741	0.957	3.168	0.070	—	—	—	—	2.144	1.236	3.720	0.007

**Table 2:** Immune-related genes correlated with ADGRD1.

Gene	Cor-LUAD	Pval-LUAD	Cor-LUSC	Pval-LUSC	Gene	Cor-LUAD	Pval-LUAD	Cor-LUSC	Pval-LUSC	Gene	Cor-LUAD	Pval-LUAD	Cor-LUSC	Pval-LUSC
A2M	0.332	5.604E-15	0.597	9.437E-50	FCGRT	0.250	6.557E-09	0.446	8.001E-26	PLAAT4	-0.253	3.926E-09	0.283	1.165E-10
ACKR1	0.225	1.902E-07	0.457	2.867E-27	FGF9	0.247	9.623E-09	0.333	1.808E-14	PPIA	-0.323	3.147E-14	-0.255	7.379E-09
ADM	-0.403	6.59E-22	-0.294	2.059E-11	FIGNL2	0.207	1.743E-06	0.264	2.05E-09	PRDX1	-0.214	6.983E-07	-0.241	4.657E-08
AGER	0.305	9.124E-13	0.544	6.278E-40	FLT4	0.226	1.712E-07	0.565	1.307E-43	PSMC1	-0.291	9.312E-12	-0.233	1.35E-07
AGTR2	0.227	1.474E-07	0.514	4.688E-35	FOS	0.307	6.232E-13	0.223	4.611E-07	PSMC2	-0.432	2.61E-25	-0.296	1.294E-11
ANGPT1	0.302	1.543E-12	0.374	4.437E-18	GDF10	0.391	1.271E-20	0.586	1.918E-47	PSMC5	-0.240	2.372E-08	-0.210	2.028E-06
ARRB1	0.504	3.561E-35	0.553	1.614E-41	GPI	-0.316	1.215E-13	-0.263	2.183E-09	PSMD11	-0.330	8.717E-15	-0.236	8.561E-08
BID	-0.399	1.709E-21	-0.225	3.389E-07	GREM2	0.208	1.455E-06	0.360	9.315E-17	PSMD14	-0.360	1.595E-17	-0.244	3.201E-08
BIRC5	-0.518	1.817E-37	-0.279	1.958E-10	HAMP	-0.216	5.735E-07	0.328	4.868E-14	PSMD2	-0.412	5.873E-23	-0.291	3.107E-11
BMP2	0.367	3.147E-18	0.312	9.038E-13	HDGFL3	-0.209	1.33E-06	-0.260	3.675E-09	PTAFR	-0.260	1.488E-09	0.232	1.593E-07
BMP5	0.222	2.663E-07	0.404	4.24E-21	HLA-B	-0.286	2.169E-11	0.219	7.016E-07	PTGDR2	0.249	7.074E-09	0.271	6.845E-10
BMP6	0.334	3.925E-15	0.305	2.867E-12	HLA-C	-0.250	6.074E-09	0.225	3.822E-07	PTGER3	0.259	1.579E-09	0.214	1.388E-06
BRD8	0.263	8.989E-10	0.228	2.597E-07	HLA-F	-0.228	1.312E-07	0.238	7.27E-08	PTH1R	0.297	3.337E-12	0.463	5.183E-28
C3	0.253	3.888E-09	0.405	3.194E-21	HLA-G	-0.200	3.617E-06	0.282	1.354E-10	RAC1	-0.382	9.304E-20	-0.257	5.493E-09
C5	0.247	8.806E-09	0.425	2.271E-23	IL11RA	0.245	1.314E-08	0.264	1.866E-09	RAET1L	-0.265	6.876E-10	-0.248	1.939E-08
CALCA	0.247	9.685E-09	0.316	4.554E-13	IL12B	0.242	1.953E-08	0.273	5.352E-10	RARG	-0.202	3.052E-06	-0.261	2.98E-09
CALCRL	0.274	1.631E-10	0.313	7.24E-13	IL1A	-0.324	2.678E-14	-0.230	2.04E-07	RNASE2	-0.211	1.043E-06	0.271	7.165E-10
CAT	0.329	9.807E-15	0.225	3.516E-07	IL20RB	-0.278	7.988E-11	-0.268	1.157E-09	RORC	0.435	1.069E-25	0.422	4.259E-23
CCL14	0.245	1.333E-08	0.530	1.404E-37	IL2RA	-0.211	1.018E-06	0.231	1.793E-07	S100A11	-0.339	1.358E-15	-0.399	1.322E-20
CCL16	0.216	5.505E-07	0.285	7.728E-11	IL33	0.228	1.194E-07	0.223	4.376E-07	S100A16	-0.234	5.668E-08	-0.291	3.242E-11
CCL2	-0.234	5.563E-08	0.338	6.805E-15	IL36RN	-0.256	2.569E-09	-0.205	3.672E-06	S100A2	-0.284	3.293E-11	-0.350	6.423E-16
CCL4	-0.279	7.328E-11	0.232	1.516E-07	IL6R	0.443	1.126E-26	0.222	4.988E-07	S100A8	-0.328	1.248E-14	-0.207	3.093E-06
CD14	-0.223	2.521E-07	0.308	1.713E-12	IL6ST	0.355	4.807E-17	0.412	6.431E-22	S1PR1	0.273	2.014E-10	0.588	7.096E-48
CD72	-0.233	6.658E-08	0.367	2.112E-17	INSR	0.273	1.964E-10	0.210	2.225E-06	SCTR	0.266	6.059E-10	0.477	7.084E-30
CD81	0.318	7.312E-14	0.341	4.038E-15	KL	0.407	2.141E-22	0.419	1.154E-22	SEM1	-0.350	1.466E-16	-0.342	3.19E-15
CD86	-0.208	1.466E-06	0.323	1.292E-13	LCN10	0.225	1.847E-07	0.341	4.456E-15	SEMA3G	0.362	8.943E-18	0.492	7.027E-32
CD8A	-0.221	3.036E-07	0.260	3.659E-09	LCN6	0.256	2.425E-09	0.342	3.107E-15	SEMA5A	0.219	3.894E-07	0.222	5.289E-07
CD8B	-0.247	9.988E-09	0.211	1.803E-06	LEFTY2	0.244	1.482E-08	0.427	1.398E-23	SEMA7A	-0.311	2.866E-13	0.222	4.976E-07
CMA1	0.203	2.786E-06	0.311	1.153E-12	LIFR	0.254	3.411E-09	0.326	6.859E-14	SFTPA1	0.340	9.734E-16	0.412	6.709E-22
CMTM1	-0.255	2.83E-09	0.254	8.211E-09	LTBP2	0.234	5.399E-08	0.370	1.103E-17	SFTPA2	0.337	1.869E-15	0.401	8.955E-21
CMTM3	-0.327	1.343E-14	0.278	2.404E-10	LTBP3	0.296	4.495E-12	0.286	6.773E-11	SFTPD	0.404	4.313E-22	0.462	6.77E-28
CMTM4	0.220	3.571E-07	0.240	5.527E-08	MASP2	0.209	1.389E-06	0.262	2.656E-09	SLIT2	0.266	5.744E-10	0.272	5.897E-10
CRLF1	0.405	3.846E-22	0.202	5.097E-06	MPL	0.240	2.565E-08	0.244	3.316E-08	SSTR1	0.202	3.134E-06	0.411	7.323E-22
CRLF2	-0.218	4.713E-07	0.332	2.326E-14	NCK1	-0.344	4.348E-16	-0.262	2.565E-09	STING1	0.201	3.51E-06	0.266	1.455E-09
CSF3R	0.221	2.966E-07	0.436	1.023E-24	NFATC3	0.260	1.37E-09	0.224	4.278E-07	TEK	0.306	6.746E-13	0.573	4.297E-45
CSRP1	0.234	5.414E-08	0.283	1.067E-10	NOD1	0.363	8.42E-18	0.356	1.891E-16	TFRC	-0.355	4.43E-17	-0.238	7.08E-08
CTSE	0.233	6.354E-08	0.382	8.014E-19	NPR1	0.320	5.236E-14	0.578	6.268E-46	TGFBR2	0.271	2.624E-10	0.480	2.965E-30
CTSG	0.204	2.499E-06	0.348	1.079E-15	NR2C2	0.285	2.924E-11	0.221	5.53E-07	THPO	0.248	7.676E-09	0.258	4.854E-09
CTSL	-0.284	3.458E-11	0.321	1.833E-13	NR2F1	0.298	3.082E-12	0.428	8.619E-24	THRA	0.279	7.652E-11	0.218	8.219E-07
CXCL16	0.247	8.845E-09	0.321	1.708E-13	NR3C2	0.285	2.643E-11	0.337	8.434E-15	TIE1	0.255	2.922E-09	0.572	5.833E-45
CXCL2	0.202	3.125E-06	0.405	3.295E-21	NR4A1	0.419	9.968E-24	0.414	3.238E-22	TMPRSS6	0.291	1.081E-11	0.343	2.91E-15
DES	0.246	1.021E-08	0.534	2.652E-38	NR4A2	0.294	5.948E-12	0.262	2.652E-09	TNFRSF10C	0.291	9.581E-12	0.366	2.775E-17
DLL4	0.274	1.533E-10	0.576	1.44E-45	OGN	0.253	4.026E-09	0.458	2.305E-27	TNFRSF19	0.247	1.001E-08	0.264	1.841E-09
EBI3	-0.226	1.598E-07	0.249	1.653E-08	ORM1	0.287	2.11E-11	0.358	1.381E-16	TNFSF13	0.203	2.545E-06	0.395	3.436E-20
EDNRB	0.401	9.079E-22	0.583	7.348E-47	ORM2	0.317	9.988E-14	0.409	1.369E-21	TNFSF13B	-0.209	1.316E-06	0.337	9.096E-15
ELN	0.330	7.533E-15	0.466	2.382E-28	PAK1	-0.274	1.519E-10	-0.217	9.534E-07	VEGFD	0.371	1.244E-18	0.517	1.213E-35
EPOR	0.261	1.208E-09	0.360	8.588E-17	PAK2	-0.336	2.23E-15	-0.217	9.421E-07	VIP	0.214	7.19E-07	0.299	7.782E-12
FAM3C	-0.369	1.892E-18	-0.218	8.726E-07	PDCD1	-0.244	1.533E-08	0.331	2.781E-14	VIPR1	0.422	3.712E-24	0.259	4.14E-09
FCER1G	-0.234	5.589E-08	0.330	3.144E-14	PGC	0.523	2.565E-38	0.525	6.936E-37	WFDC2	0.257	2.318E-09	0.203	4.817E-06
FCGR3A	-0.237	3.957E-08	0.325	8.087E-14	PGR	0.224	1.965E-07	0.401	9.386E-21					

**Table 3:** Correlation analysis between ADGRD1 and predicted microRNAs.

Cancer	MicroRNA	Gene	Cor	CorPval	LogFC	diffPval
LUAD	Hsa-miR-142-5p	ADGRD1	-0.231	1.311*E* − 07	1.138	2.958*E* − 04
LUSC	Hsa-miR-93-5p	ADGRD1	-0.267	3.743*E* − 09	1.024	2.908*E* − 13
	Hsa-miR-17-5p	ADGRD1	-0.250	3.508*E* − 08	1.186	4.215*E* − 17

## Data Availability

The original data in this research could be obtained from public databases. All data supporting the findings of this study are included in the article.

## Supplementary Materials

Supplementary Materials Supplementary Table 1: the ESTIMATE algorithm scores of ADGRD1 across cancers. Supplementary Figure 1: relationship between ADGRD1 gene expression and infiltrating levels of immune cells in different cancers.
