# Supplementary material for: ADGRD1 as a Potential Prognostic and Immunological Biomarker in Non-Small-Cell Lung Cancer
Source: Biomed Res Int. 2022 Nov 22;2022:5699892. doi: 10.1155/2022/5699892 (PMC9708333; doi:10.1155/2022/5699892)
Supplement: Supplementary Materials — Supplementary Table 1: the ESTIMATE algorithm scores of ADGRD1 across cancers. Supplementary Figure 1: relationship between ADGRD1 gene expression and infiltrating levels of immune cells in different cancers. [file 5699892.f1.zip › Supplementary Table 1.docx]

**Table S1** The ESTIMATE algorithm scores of ADGRD1 across cancers

| CancerType | Gene | StromalScore | ImmuneScore | ESTIMATEScore |
| --- | --- | --- | --- | --- |
| ACC | ADGRD1 | 0.015 | < 0.001 | < 0.001 |
| BLCA | ADGRD1 | < 0.001 | < 0.001 | < 0.001 |
| BRCA | ADGRD1 | < 0.001 | < 0.001 | < 0.001 |
| CESC | ADGRD1 | < 0.001 | < 0.001 | < 0.001 |
| CHOL | ADGRD1 | 0.004 | 0.110 | 0.025 |
| COAD | ADGRD1 | < 0.001 | < 0.001 | < 0.001 |
| DLBC | ADGRD1 | 0.005 | 0.364 | 0.224 |
| ESCA | ADGRD1 | < 0.001 | < 0.001 | < 0.001 |
| GBM | ADGRD1 | 0.022 | 0.235 | 0.116 |
| HNSC | ADGRD1 | < 0.001 | < 0.001 | < 0.001 |
| KICH | ADGRD1 | < 0.001 | < 0.001 | < 0.001 |
| KIRC | ADGRD1 | < 0.001 | < 0.001 | < 0.001 |
| KIRP | ADGRD1 | < 0.001 | < 0.001 | < 0.001 |
| LAML | ADGRD1 | < 0.001 | < 0.001 | < 0.001 |
| LGG | ADGRD1 | 0.014 | 0.246 | 0.850 |
| LIHC | ADGRD1 | 0.009 | 0.134 | 0.025 |
| LUAD | ADGRD1 | 0.567 | 0.007 | 0.190 |
| LUSC | ADGRD1 | < 0.001 | < 0.001 | < 0.001 |
| MESO | ADGRD1 | 0.031 | 0.858 | 0.200 |
| OV | ADGRD1 | < 0.001 | 0.027 | 0.308 |
| PAAD | ADGRD1 | < 0.001 | < 0.001 | < 0.001 |
| PCPG | ADGRD1 | < 0.001 | 0.008 | < 0.001 |
| PRAD | ADGRD1 | < 0.001 | < 0.001 | < 0.001 |
| READ | ADGRD1 | < 0.001 | < 0.001 | < 0.001 |
| SARC | ADGRD1 | < 0.001 | 0.016 | < 0.001 |
| SKCM | ADGRD1 | < 0.001 | < 0.001 | < 0.001 |
| STAD | ADGRD1 | < 0.001 | < 0.001 | < 0.001 |
| TGCT | ADGRD1 | 0.527 | 0.190 | 0.109 |
| THCA | ADGRD1 | 0.003 | < 0.001 | 0.001 |
| THYM | ADGRD1 | < 0.001 | 0.907 | 0.008 |
| UCEC | ADGRD1 | < 0.001 | 0.037 | < 0.001 |
| UCS | ADGRD1 | 0.367 | 0.181 | 0.723 |
| UVM | ADGRD1 | 0.151 | 0.907 | 0.563 |
