# Supplementary figures and images for: ADGRD1 as a Potential Prognostic and Immunological Biomarker in Non-Small-Cell Lung Cancer
Source: Biomed Res Int. 2022 Nov 22;2022:5699892. doi: 10.1155/2022/5699892 (PMC9708333; doi:10.1155/2022/5699892)

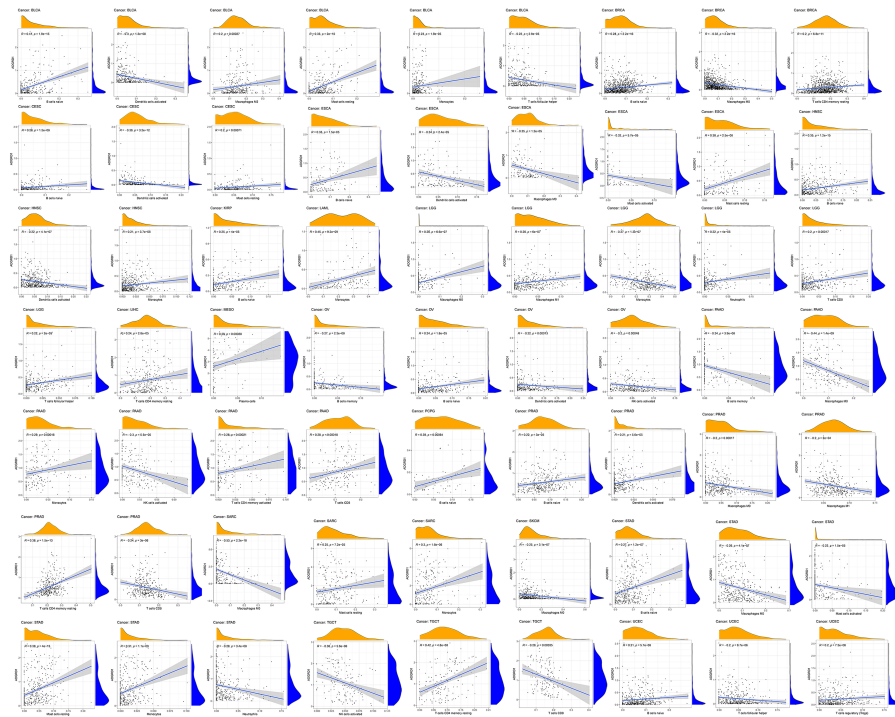

Supplement: Supplementary Materials — Supplementary Table 1: the ESTIMATE algorithm scores of ADGRD1 across cancers. Supplementary Figure 1: relationship between ADGRD1 gene expression and infiltrating levels of immune cells in different cancers. [file 5699892.f1.zip › Supplementary Figure 1 (1).pdf]
